# Neuromimesis: Picturing the Humanities Picturing the Brain

**DOI:** 10.3389/fnint.2022.760785

**Published:** 2022-10-14

**Authors:** Cate Reilly

**Affiliations:** Program in Literature, Trinity College of Arts and Sciences, Duke University, Durham, NC, United States

**Keywords:** Ramón y Cajal, cognitive media theory, art and science, neurohumanities, neuropsychoanalysis, neuroimaging (anatomic and functional), mimesis

## Abstract

What do neuroscientific visualizations of mental functioning depict? This article argues that neuroscientific imaging from Santiago Ramón y Cajal’s pen and ink drawings onward falls within the mimetic tradition, that dealing with the artistic representation of reality. Cajal’s iconic images of pyramidal neurons and glial cells surprisingly suggest a non-realist approach to picturing the brain and the mind that opens a new methodological link between humanities and neurosciences. In it, aesthetic works offer a perspective on mimetic practices in neurosciences, providing insight into representational strategies that make otherwise invisible psychic phenomena observable. This approach draws needed attention to the role of metaphor in neuroscientific research. It also reimagines how interdisciplinary scholarship might engage with works of art. While it is a common practice to read humanities objects featuring the brain and/or the mind in terms of their neuroscientific content, films like *The Headless Woman* (La mujer sin cabeza, dir. Martel, 2008), explored here, show that doing so can easily inhibit interpretations with greater explanatory bearing. Together, Cajal’s images and Martel’s film help elaborate a fresh methodological paradigm—distinct from that of neuropsychoanalysis—that situates aesthetic objects as a long-neglected tool for studying the brain by virtue of (not despite) their imaginative investments.

“There are hazy pictures and even actual gaps in the cinematograph of memory, […] when the attention, like photography on a dull day, had not enough energy to impress the film of the brain [*la película cerebral*]” (Santiago Ramón y Cajal^1^).

“In the [close-up], we recognize the mental act of attending, in the [cut-back] we must recognize the mental act of remembering” (Hugo Münsterberg).

“[…] we should picture [*vorstellen*] the instrument which carries out our mental functions as resembling [*wie etwa*] a compound microscope or photographic apparatus, or something of the kind [*u. dgl*.]” (Sigmund Freud).

## Introduction

### Seeing Is Believing

These three quotations, made by the fin-de-siècle founders of neuroscience, industrial psychology, and psychoanalysis respectively, all address a unique problem faced by early researchers on cognition: how to picture mental life? Because invisible, mental functioning posed a unique challenge to 19th- and 20th-century scientists seeking to objectively study it, as it continues to at present. Contemporaneous approaches that set out to resolve the problem shared a common, if paradoxical, goal of representing the invisible. The issue notoriously dogged Freud as he shifted from an anatomical account of the mind to a psychodynamic one out of concern that Theodor Meynert and Carl Wernicke’s association psychology could not adequately localize “psychic elements” (*die psychischen Elemente*) simply by suggesting that the ends of nerve fibers were “immersed in the psyche” (Freud, 1953, p. 55). Freud’s speculations on the potential role of “φ, ψ, ω systems” in determining everything from memory to perception, creation of a topographical model of the mind, and concept of the mystic writing pad, all attempted to produce a more plausible structural account of psychological processes (Freud, 1961, 1966).

The problem similarly motivated late 19th-century psychologists, psychiatrists, and neurologists across Europe and North America to apply the new medium of photography as a means of picturing mental health and illness. From French neurologist’s Jean-Martin Charcot’s pictures of patients at the Salpêtrière in Paris (published in the *Iconographie photographique de la Salpêtrière*, 1876–1880) to British Francis Galton’s eugenically inspired composite photographs (claiming to show various species of human nature), turn of the century research regarded the camera as a way of visually pinning down the ethereal world of the mind. As in phrenology, researchers at the time sought a correlation between physical externality and psychological internality. Even Charles Darwin connected facial expressions with mental states, proceeding on the assumption that “certain states of mind” could be tracked on the body’s surface however imperceptible they might be in and of themselves (Darwin, 1872; Gilman, 1982).

Taking a different approach to representing the invisible, Leipzig psychologist Wilhelm Wundt used empirical measurement to depict mental functioning beginning in the 1860s. Wundt and his students treated “sensations” (*Empfindungen*) as calculable quantities and relied on graphs, charts, and tables to express the mind’s workings, building on research that psychophysicist Gustav Theodor Fechner had done 10 years earlier. The difficult problem of how to represent the mind even inspired Harvard psychologist Hugo Münsterberg to hypothesize in 1917 that popular cinema’s appeal lay in its ability to replicate spectators’ “mental processes” on screen. Close-ups approximated the mental act of paying attention. Flashbacks were like memory.

Arguably one of the best-known and most enduring efforts to visualize mental functioning is the work of Spanish neuroanatomist Santiago Ramón y Cajal (1852-1934). Cajal’s discovery of neurons as the basic biological unit of the nervous system in the 1890s, supported by his neural drawings, is not only the basis for neuron doctrine but in many accounts the neurosciences as such. His renowned pen and ink images of Purkinje cells and pyramidal neurons (which he deemed the brain’s “psychic cells”), alongside numerous others, were key to disproving reticular theory or the idea that the nervous system was composed of an undifferentiated mesh, not discrete cells. Although Cajal’s drawings initially sparked some controversy, both his images and neuron doctrine were quickly accepted by the international scientific community as keystones in the study of the brain, a position they have retained virtually without contestation since (DeFelipe and Jones, 1992, p. 244). More than a century after their creation, these drawings continue to be actively used in neuroscientific pedagogy and research. They are regarded as keenly observed illustrations of brain function that also happen to be remarkably beautiful.

On the basis of the images’ influence and success, not least as the foundation for functional neuroimaging, it might seem that the quest to depict cognition drew to a close (Llinás, 2003). Far from pulling the curtain on such efforts, Cajal’s iconic illustrations paved the way for a new array of representational approaches in the coming years, as research in neuroscience continued to grapple with mind/body dualism, the “hard problem” (why consciousness exists), and a biological basis for mental illness. Whether one turns to the cognitive revolution of the 1950s and Norbert Weiner’s writing on cybernetics, which expanded on Cajal’s work by picturing the brain and mind as a kind of computer, or to the major techno-scientific advances in neuroimaging (electron microscopy, the EEG, fMRI, PET) that prompted George Bush to declare a ‘Decade of the Brain’ in 1991, the matter of how to represent mental functioning on the basis of neural activity was no less important two decades after Cajal’s death than it was five. It remains a flashpoint today. The Human Brain Project’s (HBP) billon-euro bid in 2013 to simulate the brain’s operations with supercomputers is but one in a string of latter-day efforts to concretely represent mental states and processes.^2^ These include everything from attempts to computationally reconstruct the experience of hearing to do the same for sight (Nishimoto et al., 2011) and for dreams (Horikawa et al., 2013), to Christian Herff’s efforts to read human thoughts by creating a “brain-to-text” system (Herff et al., 2015). They also encompass Mark Solms’ dual-aspect monism, which, under the heading of neuropsychoanalysis, approaches the mind-brain relationship through a statistically distributed picture of mental functioning (Solms, 2021).

The mind-brain relationship also has a prominent role in popular visual culture. A feature-length documentary in 2020 chronicling the HBP’s struggles is a tribute to the topic’s interest to general audiences (*In Silico*, dir. Nick Hutton). This film is only the tip of the iceberg, however, when it comes to the brain’s status as a common visual signifier for mental functioning. Images widely circulating in the public sphere, like the phrenological diagram in which pennies replace mental faculties on the 2016 cover of *Newsweek*, “This is your Brain on Poverty,” a tacit evocation of the fried-egg metaphor of the Reagan era’s hugely controversial war on drugs (“This is your Brain on Drugs”), serve as a testament to the issue’s longstanding sociocultural currency. The New York Times’ multi-page spread of Cajal’s drawings in 2018, excerpted from the traveling exhibition “The Beautiful Brain: The Drawings of Santiago Ramón y Cajal,” merely completes the circuit, reintroducing the source of the 21st century’s visual cult of the brain to a public already unwittingly supersaturated with its offshoots (Smith, 2018). The brain’s distinctive tangle of furrows and ridges serves as everything from a globalized icon for intelligence to a marketing logo, a meme, and a tattoo. The longstanding fetish object of B movies (*The Brain that Wouldn’t Die*, 1962; *The Man with Two Brains*, 1983) and alien species’ regular delectation of choice (*Starship Troopers*, 1997; *BrainDead*, 2016), it is the locus at which thinking attempts to gaze on itself.

Despite the volume and variety of these images assessments of just how such representations function *as* representations or—to return to the dilemma of the 1890s—how the relationship between the material substrate of mental life and cognition is shown remain few and far between. While disagreements over how to interpret particular neuroscientific images occur, to the best of my knowledge, there is no sustained practice in any discipline of inquiring into the representational and mediatory processes that enable them in the first place. The problem is two-fold. First, there is the tricky matter of how to show cognition. Absence of attention to it as a problem produces a second issue: conflation of neuroanatomical and physiological representations of the brain with the mind. This tends to preclude substantive inquiry into the former by focusing solely on the latter’s inscrutability. Disavowing representational mediation in neuroanatomy and physiology may patch over ways in which cognitive localization remains a moving target but it cannot prevent the issue.

At least part of the reason for the lack of attention to this topic has to do with the visual tradition in which Cajal’s images emerged. His drawings, printed in his neuroscientific textbooks and articles, were regarded both in his time and since as the embodiment of “mechanical objectivity” in scientific illustrative practice. This is historians of science Lorraine Daston and Peter Galison’s term for scientific images produced with the goal of eliminating “individual volition and discretion” (Daston and Galison, 2007). As such, both Cajal’s illustrations and their latter-day heirs seem veridical, as if unbiased copies of nature. The paucity of scholarship on how visual representations of the brain and the mind configure the relationship between original and image is in keeping with the assumption—supported by the scientific illustrative tradition since Leonardo da Vinci’s anatomy and Galileo Galilei’s drawings of the moon—that no such relationship exists. In the sciences, original and image are (or should ideally be) identical.

The public controversy that erupted in 1906 between Cajal and his intellectual antagonist, Italian neurologist Golgi (1843-1926), has tended to shore up this position. Golgi, who was a stalwart proponent of reticular theory, contested the objectivity of Cajal’s images up to the moment of his joint receipt of the Nobel Prize with Cajal. Standing before the audience in Stockholm, Golgi claimed Cajal’s drawings introduced spacing between neurons where none existed. He relied on this argument to charge that his co-winner’s neuron doctrine was inaccurate and that his contrary claim about the reticulum was correct (Golgi, 1906). Cajal leveled the same charge at Golgi, asserting until the end of his life that Golgi had manipulated his own drawings to mask the separation between nerve cells (Ramón y Cajal, 1933).

Both Cajal and Golgi regarded the objectivity of their images as a direct measure of their respective theories’ validity. For each, the presence of interpretive license in the other’s illustrations was tantamount to disproof of the accompanying theory as a whole. Golgi’s allegedly inaccurate depiction of the neural mesh meant that reticular theory was wrong. Cajal’s supposed illustrative distortions, which “created” spaces between neurons, meant that neuron doctrine was illegitimate. In this regard, the 1906 conflict reflects the advent of a representational regime in the sciences that correlated the absence of human intervention with fidelity to nature (Daston and Galison, 2007). The extraordinary possibilities scientific photography seemed to hold for many scientists at the time arose from the related belief that the camera could eliminate subjective bias and disclose phenomena invisible to the naked human eye: speeding bullets, the nuances of human and animal locomotion. If subjectivity was the problem, mechanical objectivity was the solution.

Nevertheless, the fact that Cajal and Golgi conflated the validity of their claims with their images’ ability to duplicate the nature world is hardly a proof for that actually being the case. Neither does it mean that even the most studiously exact image of the brain or the mind would ever be able to achieve a more than asymptotic relationship to the original (lest the image become the brain or mind itself). The conflation merely reflects the mid-19th century rise of mechanical objectivity as a representational ideal. This does not preclude the possibility that a more subjective style of representation would augment, rather than inhibit, neuroscientific research then or now, although it does make it substantially more difficult to imagine. Attending to neuroscience as part of the mimetic tradition not only raises a question about the role of creative license in neuroscientific illustration and modeling but also yields a broader line of inquiry. Given the absence of a somatically localized site of consciousness and the gap between original and representation, what do neuroscientific visualizations and models of mental functioning depict?

## Hypothesis

### From Methodological Unilateralism to Mimesis for the Neurosciences

Answering this question, or at least beginning to answer it, requires reconsidering neuroscientific images of the brain. My thought, or (to translate somewhat) my hypothesis, is that one of the most significant representational and conceptual foundations for contemporary functional neuroimaging—Cajal’s research and drawings—is far less referentially stable than hitherto understood. This is in part due to the epistemic pressure mechanical objectivity continues to exert and in part due to the related assumption that it is difficult to imagine humanities offering neurosciences much beyond their subject matter (DeVos and Pluth, 2016, p. 2). The unacknowledged polysemy in Cajal’s drawings nevertheless tacitly conditions subsequent neuroscientific research, predetermining how representations of mental functioning look rather than inquiring why they appear in a particular way.

One of the most significant challenges to considering what Cajal’s neural images show is stepping away from the retrospective narrative of his neuroscientific accomplishments. Cajal’s forceful statements about his drawings’ verisimilitude, combined with the emphasis on mechanical objectivity in his time, have led biographical, historical, and scientific scholarship on Cajal to sharply distinguish between his successful career as a neuroscientist and his lesser-known but fervent efforts to become an artist (De Rijcke, 2008; DeFelipe, 2010, 2017; Schoonover, 2010; Mariìa et al., 2015; Rinder, 2016; Newman et al., 2017). Cajal did not begin with the notion of winning a Nobel Prize in Physiology or Medicine, or even of specializing in neuroanatomy. From his earliest years, he was fixated on a different path: Cajal wanted desperately to become a painter. His efforts to forge a career in the visual arts make up the first volume of his autobiography, several hundred pages in the Spanish edition, which he augmented with examples of his own creative work. Contemporary scholastic publications and the various museum exhibitions dedicated to Cajal’s neural drawings nevertheless give this aspect of his life relatively short shrift. “The first part [of the autobiography] offers an impassioned and conflicted exploration of the role of art in his life; the second part is a calmer account of his career as a scientist,” one commentator concludes, reducing these years to a bout of youthful enthusiasm in the catalog for an exhibition nevertheless all about the profound artistry of Cajal’s scientific images (Newman et al., 2017, p. 22).

The narrative that the two halves of Cajal’s life are unrelated is also a feature of research that treats the objectivity of his neural drawings as a measure of the veracity of neuron doctrine. This has the effect of anxiously, if unwittingly, relitigating the 1906 clash by attempting to prove once and for all that Golgi was wrong (DeFelipe and Jones, 1992; Garcia-Lopez et al., 2010). The narrative is additionally sustained across the aisle in the visual arts. Presentations of Cajal’s neural drawings alongside the works of visual artists influenced by them (such as those by Surrealists Federico García Lorca, Salvador Dalí, and Yves Tanguy in the 2015 Zaragoza exhibition “The Physiology of Dreams”) tend to reinforce the notion of a fundamental divide between Cajal’s truthful neural renderings and their sur-realist counterparts. Similarly, historical scholarship noting the complementarity between Cajal’s work in the visual arts and his neuroscientific pursuits nevertheless balks at carrying this idea further to explore the implications of such porosity (Marañon, 1950; Otis, 1999; Márquez, 2004; Garcia-Lopez et al., 2010; DeFelipe, 2013, 2017; Robles, 2019). Cajal, so the story goes, may have wished to be an artist but happily became a scientist instead.

This position, however, requires maintaining an artificial distinction between the first part of his life and the second. It also necessitates subscribing to the debatable assertion that interpretive license in the neural drawings would invalidate the scientific validity of Cajal’s findings. In the same fell swoop, it pushes readers to ignore the import of artistic photography to Cajal throughout his career, discount the significance of his fantastical short stories, and disregard his penchant for anthropomorphizing neural life. So too does it bracket the impact that late 19th-century geopolitics had on Cajal’s work on neural boundaries (as Laura Otis has noted). All in the face of Cajal’s own remarks. Comments like his observation about “hazy pictures and even actual gaps in the cinematograph of memory” demonstrate ambivalence about the basic technique with which to represent mental functioning. Is it properly artistic or scientific in nature? In unlikely tandem with Freud and Münsterberg, Cajal rejects the camera and cinematograph as tools for documenting mental states. He refigures these devices instead as tropes for imagining the mind’s impalpable operations. The brain becomes a strip of film for capturing visual impressions for memory’s “cinematograph” in instances where the “light conditions” (attention) are correct. As elsewhere, the author of thousands of neuroanatomical drawings and histological preparations chooses not to rely on any of them but on an elaborate metaphor instead. Alongside Freud and Münsterburg, Cajal abandons literal application of the premiere scientific visualization devices of his era only to recast those same devices’ inner workings as metaphorical surrogates for cognitive processes.

Readings that stress the indexical nature of Cajal’s drawings miss that his neuroscientific images (and their cognitive successors) continue to beg the question of how to picture the mind rather than resolving it. Seen in light of Cajal’s early efforts to become an artist (not to mention the influence exerted on his work by the Romantic tradition and his intellectual debt to a nationalist movement in the Spanish arts), a rereading of the neural images opens the door to an anti-realist dimension of neuroscientific representation. This disturbs the dominant narrative in which subjective interpretation is synonymous with scientific illegitimacy.

The images return to a deep epistemological fissure within the conceptual history of mimesis, from the Greek *mimos* (μ~ιμ*o*ς) meaning “mime” and *mimeîsthai* (μιμε~ισθαι) meaning “to imitate, reenact, represent.” Declared objective, they remain tacitly configured by an older conflict between Platonic representational duplication and Aristotelian poetic fabulation that has structured the philosophical understanding of imitative practices since 4th century BC. While conceptual problems of mimesis have historically been limited to the arts, Cajal’s drawings illuminate the neurosciences as part of the mimetic tradition. Suspended between the impossible ideal of visual representation as the production of true-to-life copies (Plato) and a relativistic practice whose distortions enhance the realism of the object depicted (Aristotle), Cajal’s neural drawings reconfigure what it would mean to conceptualize a dialogue between humanities and neurosciences. They do so without requiring neuroscientists to part ways with materialism or humanists to sacrifice critical reading practices. The drawings open the possibility that creative objects could serve as interpretive tools by demonstrating how representations of cognition—whether in functional neuroimaging or Karl Friston’s Markov blankets—negotiate but do not escape the double-bind between mimesis as the unachievable production of identical copies and mimesis as the forging of illegitimate distortions.

Recognizing as much is important for both disciplines. It offers humanities scholars an alternative approach to the hitherto pervasive one organizing interdisciplinary work in this area for the past three decades. In it, neuroscientific research tends to be applied to humanities objects as an interpretive lens on the flawed assumption that the relationship only goes one way. Both cognitive literary criticism (Lisa Zunshine) and cognitive film theory (Noël Carroll, Ed Tan, and David Bordwell), first formulated in the 1980s and now thriving areas of scholarship with their own peer-reviewed journals, rely on neuroscientific facts to interpret literary and cinematic works (Carroll, 1983; Bordwell, 1989; Tan, 1996). Such unidirectionality is similarly a feature of publications in neurohumanities (Ortega and Vidal, 2011; Rose and Abi-Rached, 2013). These areas have made important interventions and will no doubt continue to do so. That is no reason for humanities scholars (or neuroscientists) to be limited to them.

In addition to facilitating a fresh methodological style, making space for a two-way dialogue would place studies on the mind and the brain in greater conversation with major movements in humanist thought. It would enable a hermeneutics of the brain rooted in the humanities’ ongoing analysis of the enlightenment’s and reason’s troubled legacy rather than trading humanities’ most incisive forms of analysis for the opposite epistemological perspective. By engaging a new facet of the philosophical interrogation of “naturalness,” it would also enable matters of gender, race, and class to come to the fore in mind sciences, the need for which a growing body of work has already begun to identify (Pitts-Taylor, 2012, 2016, 2019; Kraus, 2016). This would include acknowledgment that neural selfhood is at once an innately existing reality and a social product. It would thereby allow its elaboration to be placed in dialogue with the Foucaultian exploration of madness, the clinic, and biopolitics, not to mention the work of the Frankfurt School (and critical theory more broadly). It would actively engage, instead of foreclosing, the competition between a materialist phenomenology that largely dispenses with Edmund Husserl’s critique of psychological data empiricism [compare Francisco Varela’s ideas with part III B of Husserl (1970)] and research that suggests the abiding presence of the incorporeal within the material (Grosz, 2017). The ground of sciences is no more resolved for efforts to produce a phenomenology of the brain.

A two-way dialogue would, moreover, put the question of culture into play. Discussions by Kate Hayles, Mark Hansen, Luciana Parisi, and Bernard Stiegler have already foregrounded culture’s relationship to *technē*, machine intelligence, and the existence of a technological non-conscious (building on the work of Gilbert Simondon and Martin Heidegger). A mutual dialogue would enrich these discussions. Along the way, it would take up questions raised by deconstruction about the ethnocentrism and onto-theological bias of the *logos* in neurosciences for the first time. Such an inquiry might begin by noting the (fascinating but overlooked) co-emergence of Saussurian linguistics and modern mind sciences as new forms of *Naturwissenschaft* at the end of the 19th century. The philosophical take on neural plasticity is, by its own admission, ill-equipped to address this topic (Johnston and Malabou, 2013, p. 34). Finally, it would foreground aesthetic epistemology, stressing the need to engage deeply with literary texts, film, and visual art as sources of analysis rather than evidence of scientific claims. This is in line with Stathis Gourgouris’ argument about literature as a thinking tool (Gourgouris, 2003). It is similarly consistent with the claims of *Wissenspoetik*, or the poetology of knowledge. It marks a significant shift from how interdisciplinary cognitivists like Antonio Damasio and Oliver Sacks engage aesthetic objects: as illustrations of affect (Sacks, 1985). An added benefit would be to relieve humanists of the awkwardness of both disabusing entry-level students of the idea that the study of literature and art is about feelings and simultaneously upholding the premise that cognitive science is valuable to humanities because it explains why readers/viewers feel the way they do.

There are equal benefits for neurosciences. Perhaps foremost among them is access to a new tool: the ability to account for mimetic processes already conditioning day-to-day neuroscientific inquiries. This might mean accounting for neuroimaging’s mimetic complexities but is not limited to the visual sphere. Such an approach would make it possible to address the role of techno-metaphorics in neuroscience and cognitive science, present since Norbert Wiener’s cybernetics. It would thereby help tackle the impact of language and rhetoric on the types of research questions asked and conclusions drawn. The idea of neurons “firing” or “sharing information,” for example, figures biological operations in the militant language of World War II, *via* Weiner’s involvement in designing predictive control systems for anti-aircraft artillery (Wiener, 1965, pp. 5–6; Dupuy, 2009). How does representing brain function in the ballistic idiom of Allied defense tactics configure scientific understanding? Given that a study on language and rhetoric has already been conducted for molecular biology (through scholarship exploring the genesis of a “language of the living” with DNA) and theoretical physics (on racialized implications of expressions like “dark matter”), there is no reason not to extend the gesture further to neurobiology and neurophysiology (Jacobs, 1976; Kay, 2000; Prescod-Weinstein, 2021). In fact, it seems particularly crucial in light of the anthropomorphism potentially configuring discussions about the brain’s capacity to suffer, create a form, and serve as an agency of disobedience. Does the brain possess independent agency or only appear to when rhetorically allotted human characteristics? What does it mean to allot human characteristics to the organ whose successful functioning is, in many places, key to the legal definition of being alive?

A two-way dialogue would, moreover, help disentangle discussions of the brain’s physiological operations from the lexicon of imitation regularly employed to describe them. For example, studies on mirror neurons, predictive coding, and the brain circuitry grounding cognitive replications of others’ intentions all rely on prior figurative strategies pulled from the (English-language) idiom of mimesis to describe acts of perceiving and relating to the external world. They do so, however, without substantive awareness of it. Would it not be beneficial to understand precisely what it means to say that mirror neurons “*represent* [an] observed action” in this context, as reiterated in a breakthrough study on the topic (Rizzolati et al., 1996)? And, indeed, to examine how the rise of English as a globalized scientific *lingua franca* may be responsible for determining that meaning, flattening the complexities of *Vertretung*, *Darstellung, representatio, représentation*, and other untranslatables?

Finally, it would offer the neurosciences a more capacious understanding of aesthetic objects. Such an approach would prevent the looping phenomenon in which claims about the brain’s representational abilities become the basis for describing what aesthetic objects do. To take the example of mirror neurons, it would permit scholars to avoid confusing the activities undertaken by mirror neurons with aesthetic objects’ distinct representational processes, a conflation common in explanations of cinema as embodied simulation (Gallese and Guerra, 2020). It would also facilitate new questions. A purely colloquial idea of representation obscures the distinction between representation in the sense of a vehicle (the oil and canvas, in the case of a painting) and representation in the sense of a reproduced event/object/person (the painting’s subject matter) (Heidegger, 2008). Are mirror neurons the vehicle, content, both, or neither? As discussed below, two-way engagement would further provide a fresh model for imagining the bridge between “objective” and “subjective” analyses that is different in nature from (but in certain ways complementary with) Mark Solms’ bid to repair the break between Freudian and biomaterialist accounts of cognition by way of neuropsychoanalysis.

To be sure, Cajal is not the only way to start a discussion about the mimetic dimension of neurosciences. His work is a useful point of entry, however, insofar as the objectivity of his drawings has been a keystone of neuroscience for over a century and exerted substantive influence on subsequent neuroimaging practices. Unlike with Freud’s oeuvre, there is also a consensus in the contemporary neuroscientific community that Cajal’s research is scientifically rigorous. Showing that Freud’s diagrams possessed a subjective dimension would not surprise many, as that is already the overriding conclusion about the psychoanalysis made by received wisdom and pop psychology. It has been driven in no small part by the field’s decline as a recognized medical practice. Showing that Cajal possesses the same subjective dimension is a different story.

## Methods

### Cajal’s Neural Landscapes

Cajal’s autobiography, *Recollections of My Life* (Ramón y Cajal, 1917) spends much of the first volume (some three hundred pages) recounting its author’s artistic endeavors and frustrated creative hopes. Lavishly illustrated with Cajal’s own drawings and photographs, the *Recollections* contains extensive, detailed prose descriptions of the architecture of the Aragon region of Spain where Cajal was born and educated and that of other parts of the country where he traveled or held positions. By his own account, art was his first love and obsession:

When I was about 8 or 9 years old […] I already had an irresistible mania for scribbling on paper, drawing ornaments in books, daubing on walls, gates, doors, and recently painted facades […]. A smooth white wall exercised upon me an irresistible fascination. Whenever I got hold of a few cents I bought paper or pencils; but as I could not draw at home because my parents considered painting a sinful amusement, I went out into the country, and […] drew carts, horses, villagers, and whatever objects of the countryside interested me. Of all these I made a great collection, which I guarded like a treasure of gold (Santiago Ramón y Cajal, *Recollections of my Life*, translated by E. Horne Craigie, p. 36).

Too poor to purchase paints, he recounts coloring early drawings by soaking colors out of books of cigarette paper, an effort that testifies as much to youthful ingenuity as the determination with which he pursued this proscribed amusement (Ramón y Cajal, 1996, p. 36).

To his father’s disappointment, Cajal’s determination to draw apparently did not lessen over time. He claims that until his 20s, he and Justo Ramón Casasús (a physician) were locked in an unrelenting battle over his madness for drawing. His father believed it could lead only to poverty, not least because Cajal allegedly lacked a shred of talent. According to Cajal, his father was determined that he and his brother would pursue careers in medicine. Cajal’s father’s remonstrations and unwillingness to accept the praise heaped on Cajal by the drawing instructors with whom he studied during his schooling had no effect. Cajal describes remaining an inveterate caricaturist of his teachers, whose frequent rebukes did little more than spur him on.

Outside of the classroom, he took pictorial inspiration from nature and continued to make very ready use of blank walls. His autobiography recounts efforts to create a pictorial dictionary of colors accompanied by drawings of corresponding natural objects. Because he could not afford flowers, he stole them (Ramón y Cajal, 1996, p. 93). An illustrated book written in the style of Jules Verne (Cajal’s fictitious traveler explores the anatomy of giant aliens on Jupiter) was followed by extensive practice under the guidance of painter and professor Leon Abadías y Santolaria. Supervised by his instructor, he copied the drawings of Greek masters and the Madonnas of Rafael. Cajal also experimented with water color, aquarelle, and oil.

All of this is, of course, Cajal’s story about himself. One need not take his statements at face value, however, to conclude that Cajal regarded this era of his life as important. Whatever the historical accuracy of volume one of the *Recollections*, it documents Cajal’s desire for readers to know about his artistic background and empathize with his frustrated efforts to pursue a career as a painter. The first volume of the *Recollections* also makes clear that strict verisimilitude was something of an anathema to Cajal. His main reaction upon reading *Don Quixote* as a young person was apparently despair at Cervantes’ excessive realism. The short stories Cajal composed later in life are notable for their fantastical scenarios. His protagonists include a man with eyes like microscopes, another who creates a serum to eliminate sin, and a third who contrives to age young women (while preserving their mental capacity) so as to combat the “dangers” of female sexual appeal (Ramón y Cajal, 2001). Instead of writers like Cervantes, Cajal acknowledges being inspired at an early age by the works of Romantic authors: Victor Hugo, François-René de Chateaubriand, Lord Byron, and José de Espronceda. He even claims to have borne the stamp of the Romantic movement’s melodramatic fatalism and obsession with a sublime nature—not to mention its valorization of an intensely subjective heroic solitude—for much of his life (Ramón y Cajal, 1996, p. 101).

Packaged definitions of Romanticism and Enlightenment tend to separate the two on the basis of the Romantic movement’s rejection of Enlightenment rationality. Allegedly, Romanticism replaced the *ratio* with freedom of expression, emotional directness, the unbounded nature of subjective imagination, and appreciation for flights of extreme affective intensity. Whereas the period in Western Europe between the 1760s and 1790s had been dominated by aesthetic focus on an ordered and impersonal world, this purportedly changed between the 18th and 19th centuries, as artists and writers began to valorize psychological interiority and cultivate an interest in irrationality. Scholars in the humanities have demonstrated, however, that such periodization misses the complexity of Enlightenment and Romanticism’s relationship. Both Cajal’s explicit comments about the influence of Romanticism on his life, as well as the style in which his autobiography is written, are a testament to the Romantic movement’s enduring impact on him but hardly in the form of a binary turn from Enlightenment. His writing bears out the interwoven nature of reason and romance, the fact that, as Deborah White remarks, “Romantic discourse emerges in the Enlightenment ‘context’ of skepticism and critique” rather than in contradiction to it (White, 2000, p. 10). Neuroscientific research continuing to tout the hard distinction between objectivity and subjectivity thus unwittingly remains trapped within a rejection of a cliché about Romanticism that is at best of dubious validity.

**FIGURE 1:**
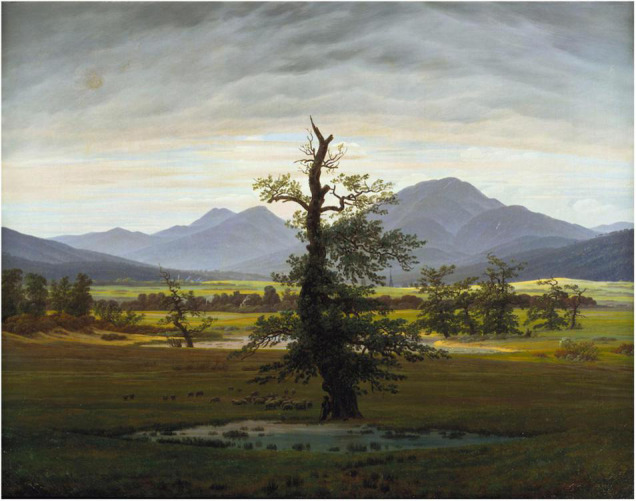
Caspar David Friedrich, *Der einsame Baum* (The Solitary Tree), 1822, oil on canvas, 21.6 × 27.9 in (55 × 71 cm), Alte Nationalgalerie, Berlin, http://commons.wikimedia.org/wiki/File:Caspar_David_Friedrich_-_Der_einsame_Baum_-_Google_Art_Project.jpg.

**FIGURE 2:**
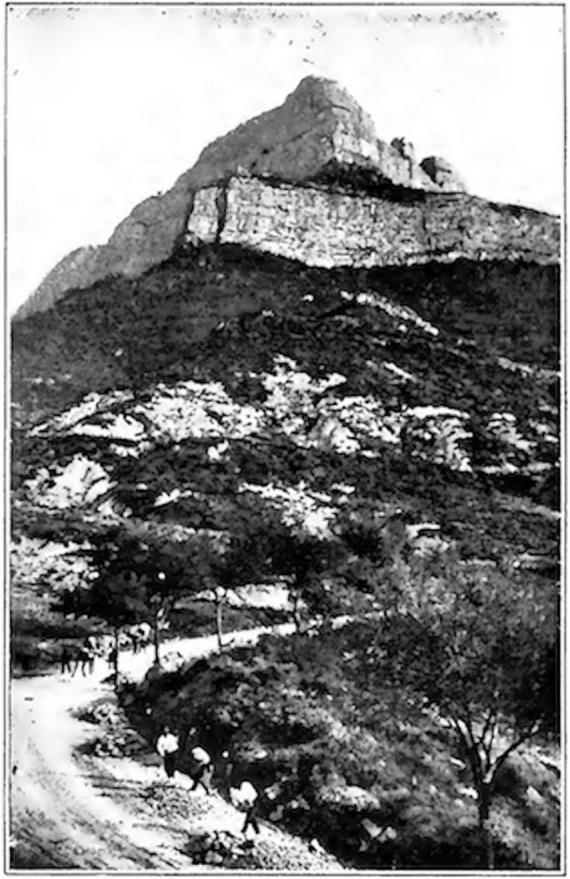
Cajal’s photograph of Monte Oruel (in Jaca, Spain) seen from the west. Digital reproduction of Plate 7 in Santiago Ramón y Cajal, *Recuerdos de mi vida*, Volume 1 (Imprenta y libería de Nicolás Moya, Madrid, 1917). Available at http://www.gutenberg.org/ebooks/58331.

Cajal’s prose is indeed strewn with passionate, grandiose descriptions that borrow from the Romantic canon of aching desire and crushing despair. He discusses attempting to ascend the Coll de Ladrones in the Pyrenees in pursuit of a view of “crystal and placid lakes bordered by lofty cliffs of painted rock over the steps of which there fling themselves rainbow cascades,” not unlike Byron’s Childe Harold (Ramón y Cajal, 1996, p. 62). Commenting on his delight in classical art, he writes of having been long “intoxicated” with the “aesthetic instinct, which at last quenched its thirst for the ideal in the pure stream of classic beauty,” a sentence no less replete with subjective fervor and rarefied abstraction in English than in Spanish (129–130). These sorts of descriptions, as well as his landscape photographs from the 1870s and 1880s featuring the transcendent intensity of the Aragonese mountains and mysterious depths of the forest, replay the Romantic preoccupation with the immensity of nature as a trope for man’s subjective life (Figures 1, 2). They resonate particularly with German painter Caspar David Friedrich’s work (Villalabos, 2013, p. 192). In Friedrich’s well-known painting, “Wanderer above the Sea Fog” (1818), a lone man stands on a rocky outcrop with his back to the viewer, gazing philosophically into the blue-gray distance as fog swaths the jagged peaks surrounding him. In an early oil painting, Cajal depicts a female figure lying on the beach, her face turned inscrutably upward as huge waves crash at the foot of a craggy precipice that could be pulled straight from “Wanderer.” Cajal’s photographs of the Aragonese landscape even resemble Friedrich’s own vistas (Figures 1, 2).

Cajal was also significantly influenced by the literary movement known as the Generation of 1898, whose aesthetic principles were formed in reaction to Spain’s declining fortunes (Ramón y Cajal, 1996, pp. 467–468). These reached a nadir with the Spanish loss of colonial holdings in Cuba, Puerto Rico, Guam, and the Philippines during the Spanish-American War of 1898. Regenerationist Spanish writers like Azorín (José Martin Ruiz, 1873-1967) and Miguel de Unamuno (1864-1936) deliberately turned to the Spanish landscape in response, developing it in their fiction as an emotionally charged and nationally inflected space that spoke to the glory of an otherwise languishing Spain (Villalabos, 2013, p. 193). As Otis has shown, a great deal of biology in the 19th century regarded the self-enclosed unit of the cell as a surrogate for contemporaneous geopolitical struggles (Otis, 1999). According to Otis, Cajal’s personal views on individual “willpower, creativity, and regeneration,” combined with his sociopolitical anxieties about the *xenos* in Spain, played a key role in his finding that Golgi’s reticular theory was erroneous (64). Only an account that showed the existence of autonomous cellular boundaries, as fixed as those of nation-states and persons, could explain the composition of the nervous system. In Otis’ telling, Cajal’s political perspective informed his neural biology. If national borders and individuals needed stalwart defense against dangerous external forces liable to overrun their naturally discretized existence, neural life must be structured similarly.

In consequence, Cajal’s claim of complete “fidelity to nature” in his neuroscientific work is more complex than it seems. In addition to being the source of immutable laws, “nature” also functioned as the externalization of first-person experience (as per the Romantic tradition) and an *Ersatz* for anxieties over geopolitical boundaries (as per the Regeneration movement and Cajal’s qualms concerning Spanish territorial sovereignty). It was also the site of some of Cajal’s most expansive metaphors. Neurology was a “garden” filled with “butterflies of the soul” (neurons), or populated by dense neural “forests” that could be readily admired for their branching dendritic “arborealizations.” Indeed, as Cajal’s contemporaries regularly observed, his scientific thinking had an intensely anthropomorphic streak. Cajal proceeded as if “the microscopic scene” were “inhabited by beings that felt, did, hoped, and tried even as we do” (Cannon, 1949, p. xiv). Describing reproductive biology, Cajal writes in a gendered formulation of the “strongest and most fortunate sperm” triumphantly “rend [ing] the mysterious veil of the vitelline membrane and, losing its degrading tail, unit[ing] itself at last in sublime conjugation with the female nucleus” (Ramón y Cajal, 1996, p. 297). In a less familiar variation, the metaphor recurs in his characterization of neural life, where synapses become the orgasmic “final ecstasy” of neurons’ “protoplasmic kiss” (Ramón y Cajal, 1996, pp. 363, 373). The inverse gesture, a representation of human love as identical to physiology, reappears in his fiction, complicating whether Cajal’s stories narrativize biology or his biology is shaped by narrative.

**FIGURE 3:**
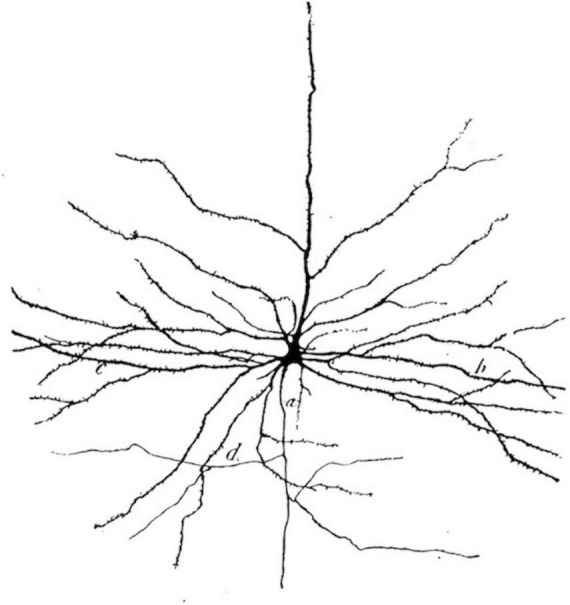
Cajal’s illustration of a giant pyramidal neuron in a 30-year-old male, as it appears in his textbook. Digital reproduction of Figure 690 in Santiago Ramón y Cajal, *Textura del sistema nervioso del hombre y los vertebrados*, Volume 2, Pt. 2 (Imprenta y librería de Nicolás Moya, Madrid, 1904), 838. Available at http://www.google.com/books/edition/_/AUlQAQAAMAAJ?hl=en&gbpv=1.

**FIGURE 4:**
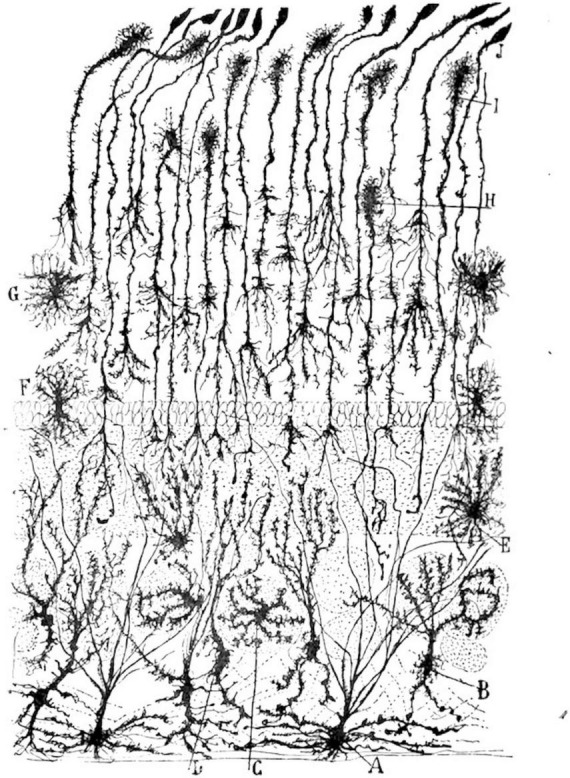
Cajal’s illustration of glial cells in the olfactory bulb of a kitten. The image resembles the forested landscapes shown in Figures 1, 2. Digital reproduction of Figure 743 in Santiago Ramón y Cajal, *Textura del sistema nervioso del hombre y los* vertebrados, Volume 2, Pt. 2 (Imprenta y librería de Nicolás Moya, Madrid, 1904), 936. Available at http://www.google.com/books/edition/_/AUlQAQAAMAAJ?hl=en&gbpv=1.

Otis’ overall argument could easily be extended to an interpretation of Cajal’s drawings. In this case, addressing what neuroscientific visualizations and models of mental life depict would mean acknowledging their intentional “mistake” of including a thematic material from outside neurosciences. It would mean seeing Cajal’s drawings as objective scientific images and nationalist Romantic landscapes (Figures 1–4). While highlighting neural discretization, these images also showcase the borders of individual neuron states (thus satisfying the need to naturalize geopolitical borders at the level of biology itself) and compensatorily conquer a “new territory” for Spain by laying claim to the brain’s *terra incognita* (thus symbolically recuperating the lost Spanish Empire). Cajal’s wishful cartography of Europe, with nations neatly secured against invasion by one another, ends up projected onto a no longer strictly metaphorical “map” of the brain. Thinking in this way reveals the creeping (and unwieldy) introversion of world space into biological interiority, in contrast to the brain’s singular isolation in a purely biological space-time cordoned off from geography.

The drawings, moreover, allow Cajal to depict strong-willed and decidedly autonomous cellular protagonists playing out amours, forging connections, or warding off threats from “outside,” much like the solitary heroes of Romantic fiction and poetry. His tendency to anthropomorphize what he saw under the microscope paradoxically ascribes psychological agency to cells responsible for it, making it difficult to distinguish Cajal’s “psychic cells” (his coinage for pyramidal neurons in the cerebrum) from those to which he figuratively attributed psychological characteristics by presenting them as human beings in miniature. Just as literary scholar Barbara Johnson points out in her analysis of anthropomorphism in poetry and fiction, Cajal’s work seems to visually repeat the definitional aporia haunting *l’homme* (man) (Johnson, 2008).

Cajal’s obsession with the structure and operations of the retina (the organ of sight) puts a fine point on his interest in mimesis, obscuring the boundary between his study on photography and the body’s anatomical equivalent of the camera.^3^ All of this makes it viable to consider the neural drawings as simultaneously objective depictions of the inner, cellular world that fully met early 20th century criteria for scientific illustration and subjective expressions of Cajal’s interest in problems of representation as it intersected with his anxieties about geopolitics and Spain’s place therein.

Otis concludes that “metaphors of invasion” played a significant role in late 19th century biology, ranging from Cajal’s work to that of figures like Rudolf Virchow, Robert Koch, and Silas Weir Mitchell. Her argument also suggests that metaphor (period) has an important role in neurosciences and not just in the 19th century. Regarded in this way, the Romantic and Regenerationist qualities of Cajal’s drawings are integral to their efficacy as objective portrayals. Presenting the brain as a Romantic landscape peopled by neural and glial protagonists is complementary with the (more scientifically accepted) strategies Cajal used to create his images: drawing from memory, combining images from different samples of brain tissue into single drawings, generalizing neural features, and adjusting his illustrations to make particular arguments (Newman et al., 2017, pp. 23–26). In each case, a departure from mechanical objectivity enhances, rather than diminishes, the scientific value of the resulting images. As Eric A. Newman, Alfonso Araque, and Janet Dubinsky tellingly write, “A single drawing by Cajal summarizes a basic principle or a sequence of events much more clearly than could be shown in dozens of photographs” (9).

### Whose Mimesis?

The notion that subjective modification heightens representational fidelity is a familiar topic in humanities, albeit one also largely restricted to it. It forms the core of the enormous body of work on mimesis, widely acknowledged as pertaining to theories of imitation and resemblance in the arts since Plato but that is also considered to lack a single, stable definition. As Gunter Gebauer and Christoph Wulf remark in *Mimesis: Culture, Art, Society*, the question “What is mimesis?” is itself invalid as it “presupposes that mimesis is a largely homogenous concept that undergoes continuous development in a historical space” (Gebauer and Wulf, 1995, p. 310). Auerbach’s (2003) *Mimesis: The Representation of Reality in Western Literature* (1946), one of the first comprehensive surveys on the topic, notoriously omits any definition of the term. Instead, Auerbach shows the changing relationship between art and reality in chapter-length studies covering everything from the New Testament to Virginia Woolf and Marcel Proust. This is similarly the conceit before which the *Mimesis* volume of Routledge’s “New Critical Idiom” series (introductory books for a general audience) must bow (Potolsky, 2006, p. 11).

One of the few points of scholastic consensus about mimesis is that its legacy has been determined by the term’s polysemy in Greek philosophy. Jacquelin Lichtenstein and Elisabeth Decultot remark, writing for the *Dictionary of Untranslatables*, that such ambiguity aligns with the dual orientation given to mimesis by Plato and Aristotle: “the opposition between a concept elaborated with reference to a pictorial model, giving mimesis the sense of ‘resemblance’ and a concept elaborated with reference to a theatrical model, giving *mimêsis* the sense of ‘representation”’ (Lichtenstein and Decultot, 2014, p. 659). The split between Plato and Aristotle, they assert, is responsible for the linguistic and cultural traditions engaging mimesis as everything from the Italian Renaissance’s *imitazione* and A. W. Schlegel’s critique of *Nachahmung* to Theodor W. Adorno and Max Horkheimer’s vital experience.

In the *Republic*, Plato famously condemns mimesis, consigning it to the realm of illusion and trickery. The well-known Allegory of the Cave in Book Ten makes a powerful case that mimesis is insidious because it is capable of producing an entirely false world and consigning human beings to intellectual darkness (the cave’s depths) by forcing them to turn away from the light of reason and truth (available outside the cave). Plato accordingly bars poets from his ideal city, claiming they are little more than clever charlatans. Aristotle, in contrast, outlines a much different position in *Poetics*. Turning to theater, Aristotle presents mimesis as a legitimate process governed by unique laws. It is through mimesis that the artist (a) employs poetic fabulation to recreate the “muthos” (plot) of an event not as the historian might but according to the horizon of possibility and (b) more generally imitates nature by engaging in acts of creation.

The division between Plato’s *Republic* and Aristotle’s *Poetics* seems to restrict mimesis to artistic, not scientific, pursuits. The *Republic* is not the only place in which Plato discusses mimesis, however. Indeed, his usage is far more heterogeneous than Aristotle’s. In fact, Plato’s *Sophist* (circa 360 BC) describes a form of mimesis that is fully compatible with true knowledge (Plato, 1921). The *Sophist* pivots around the question of how to define a sophist (and sophistry) so as to better distinguish falsity from truth in an educational context. After several unsuccessful attempts to differentiate a sophist from a philosopher and a statesman, the dialogue’s main interlocutors (Theaetetus and the Eleatic Stranger) hypothesize that a sophist must be some form of an imitator. This leads the Eleatic Stranger to distinguish between a valid practice of *mimêsis eikastikê* (roughly “likeness-making”) that reproduces the original’s proportions and colors (235d–235e) and an illegitimate practice of *mimêsis phantastikê* (roughly “appearance-making”) that reproduces objects according to how they appear from the viewer’s perspective, not as they actually are (235e–236a). On the basis of this distinction, the Eleatic Stranger proposes defining a sophist as a practitioner of *mimêsis phantastikê*, someone who produces false appearances and not true likenesses.

The difficulty is that doing so generates a paradox. It violates the pre-Socratic philosopher Parmenides’ distinction between being (or *logos* in Greek) and non-being (or *pseudos* in Greek). If a sophist generates non-knowledge, then it means that non-knowledge (a form of non-being) actually exists and Parmenides’ distinction cannot be valid. In other words, it means that non-being exists. To resolve the paradox, the Eleatic Stranger spends the remainder of the dialogue demonstrating that Parmenides must really be incorrect. German phenomenologist Martin Heidegger, whose work was heavily inspired by Plato’s *Sophist*, identifies this as a paradigmatic moment in the text. It is here that Plato demonstrates “the factual existence of non-being” (Heidegger, 1997, p. 280). Plato remarkably shows that being and non-being, that *logos* (λóγ*o*ς) and *pseudos* (ψευδ ´ης), are woven together.

While Plato derives the existence of non-being from *mimêsis phantastikê* (appearance-making), he does not limit it to appearance-making. *Mimêsis eikastikê* must therefore also contain a fragment of non-being. In this case, the difference between desirable likeness-making and undesirable appearance-making is really only of one of degree and not type. This is important because it means that the *pseudos* forms a part of *mimêsis eikastikê* and *mimêsis phantastikê* alike. *Mimêsis phantastikê* has enough of the *pseudos* to deform the relationship between image and original, while *mimêsis eikastikê*, possessing less, leaves the relationship largely intact, although not perfectly so. Heidegger recognizes as much when he remarks in his lectures on the *Sophist* that “the φ ´αντασμα in its existence as an image is *even more* not that which it poses as; in it non-being is *all the more* genuine” (Heidegger, 1997, p. 278; emphasis added).

That a grain of the *pseudos* persists in *mimêsis eikastikê*, i.e., that even legitimate mimesis is still governed by the persistence of something alien to itself, matters a great deal. It suggests that scientific representation, as a form of valid *mimêsis eikastikê*, does not entirely escape subjectivity but instead depends upon an internal play of *logos* and *pseudos*, or what Heidegger calls *Anderssein*, “being-other” to itself (Heidegger, 1997, §77β). In the *Sophist* and in *Cratylus*, Plato is careful to specify that no image, however scrupulous, is ever a perfect copy of its original lest it becomes a double (and the entire reason for speaking of *an* original and *an* image, not two originals, to disappear). This makes it plausible to imagine that the irremediable persistence of non-being (*pseudos*) within *mimêsis eikastikê* (likeness-making) aids in the construction of likeness by preventing representation from collapsing into duplication. In theory, all scientific representations are subject to some degree of mimesis. Cognitive neuroscience is a special case, however, because in it, the “being-other” of scientific representation to itself, its *Anderssein*, is exacerbated by the lack of a somatic site of consciousness to directly depict. The gap demands greater reliance on *pseudos* to overcome. The Romantic and nationalist dimensions of Cajal’s neural drawings form one expression of it.

Thinking in this way suggests that neurosciences are part of the mimetic tradition, not exceptions to it. It simultaneously reveals that fiction, subjectivity, and deformation can make up part of an accurate (i.e., knowledge-producing in the Platonic sense) scientific representation. As such, Plato’s definition of a good education, premised on the ability to distinguish truth from falsity, must include attention to mimesis in order not to risk itself becoming sophistry. (Overlooking mimesis would, Platonically speaking, be bad science.) Finally, it shows that aesthetic objects offer a valuable perspective on mimetic activity. Operating from within the sphere of a Platonic *mimêsis phantastikê*, such objects provide unique insight into the irreducible shard of the *pseudos* contained in neuroscientific imaging and modeling.

There are two important implications for research seeking to work at the juncture of humanities and neurosciences. The first, discussed in the findings below, pertains to aesthetic works containing neuroscientific content. While it is understandable that scholars from a wide variety of disciplines should wish to interpret these objects in terms of scientific claims, thinking in terms of neuromimesis suggests that not all works of art with neuroscientific content are using it in the way the neurosciences do. Focusing on scientific themes could actually be interpretively misleading. If Cajal’s images are as much about Spanish geopolitics as they are about brain cells, there is no guarantee that aesthetic works thematically concerned with the brain or the mind actually have anything to do with these topics.

Martel’s (2008) film *The Headless Woman* (*La mujer sin cabeza)*, for example, appears to be about its protagonist’s disturbed mental life. Attending only to her alleged disturbance, however, makes it impossible to grasp the film’s pointed critique of visual evidence as a basis for causal reasoning. In place of a story about the brain, Martel constructs a cinematic world in which faith in visual evidence sustains hegemonic presuppositions about gender, race, and history. A careful reading of the film not only brings out this feature but also showcases an alternative interpretive practice. It complicates neurocognitive investigations attempting to study how the brain responds to art. Martel’s film demonstrates that unless the aesthetic object in question has been carefully analyzed, any study of how the brain reacts while watching, reading, or viewing it can only hope to endlessly reproduce the initial assumptions about what that object shows or does.

The second implication is related to subjectivity’s role in the neurosciences. If subjectivity is an unavoidable component of scientific representation, how should researchers go about attending to it? Taking account of subjectivity necessitates a new way of conducting interdisciplinary scholarship. It requires prioritizing the extra-disciplinary space between fact and fiction as a site of mutual inquiry for the humanities and the neurosciences in which neither possesses mastery. While Mark Solms has made a compelling case for neuropsychoanalysis as a way to incorporate subjectivity into neurosciences, his method is of limited help in making this crucial shift.

## Findings

### Interpreting Art With Neuroscientific Themes

Argentinian feminist director Martel’s (2008) *The Headless Woman* might be a film about the murder of a child. It is unambiguously one that encourages its viewers to imagine that it is about its protagonist’s mental disturbance. As the film unfolds, however, Martel cleverly explores the limits of thematic interpretation and undoes her film’s more superficial status as a narrative primarily concerned with one person’s disorientation.

The opening shows a trio of dark-skinned, ragged adolescent boys and a dog playing on a deserted road in the rural, northwestern province of Salta, Argentina. The subsequent 87 minutes follow the bourgeois, middle-aged, light-skinned dentist Verónica (Vero) during and after she had a car accident on the same road. Vero does not get out of the car at the time of her accident, and the camera likewise remains strapped into the passenger seat. As a result, it is unclear who or what she has hit. The matter is never conclusively resolved. The film struggles to come to terms with what has (or has not) happened in the crash. This uncertainty forms its affective soundtrack, locked on repeat in all that follows. A bizarre visit to the hospital (in which Vero has X-rays taken of her head but writes her nurse’s name on the paperwork), a one-night stay in the local hotel (where she sleeps with a relative), and a fruitless attempt to go to work the next morning (Vero thinks she is a patient in her own dental clinic), all seem to indicate that she has become disorientated. The camera appears to be reconstructing her experience of bewilderment, which the film strongly suggests is the consequence of a traumatic head injury she received during the accident. As the minutes roll by, however, it also drops clues that Vero might be suffering from a psychological disturbance or struggling with a combination of physical and psychological difficulties triggered by the crash.

Whatever the source, the implication is quite clearly that there is something medically wrong with Vero. Able to function only through the ministrations of her pointedly anonymized darker- skinned servants (on whom she relies for even basic tasks), Vero’s disturbance is ironically magnified by her attendants’ pretense that nothing is wrong, even as they compensate for her lapses. Her suggested level of disorientation is severe enough to throw the entire film into disarray: suspending normal causality, shattering linear narrative, and scrambling the viewer’s attempts to distill the roughest approximation of objective reality from the protagonist’s subjective gaze.

Secondary literature in the humanities on *La mujer* overwhelmingly endorses this assertion. Multiple commentators conclude that Vero is experiencing amnesia (Quintín, 2008; Quirós, 2010; Martin, 2013; Vásquez, 2015; White, 2015), while others speculate that she has been traumatized (Sosa, 2009; Losada, 2010). Explanations additionally include that Vero is concussed (White, 2015), confused (Martin, 2013), has “lost her bearings” (Rodríguez, 2014), is plagued by a guilty conscience, or is experiencing a general psychological crisis connected with her privileged economic status in a racist neocolonial order (Losada, 2010; Vásquez, 2015). Even more diagnostically circumspect commentators conclude that Vero is, at very least, “in an altered state of mind” (Gemünden, 2019) or that the film is depicting an erratic “condition of consciousness” (Gemünden, 2019, p. 74). Catherine Malabou might characterize Vero as one of the “new wounded” (Malabou, 2012).

While no cognitive readings of the film currently exist (to the best of my knowledge), it lends quite itself readily to this style of interpretation for the same reasons that humanists identify: *La mujer* seems to be about something amiss with Vero. Adopting the neurocinematic approach outlined by Vittorio Gallese and Michele Guerra in *The Empathetic Screen* might involve exploring how camera techniques facilitate viewer empathy with Vero’s plight by engaging mirror neurons (2015, 2020). As Gallese and Guerra outline, viewers’ neural response to differing camera techniques can be measured by using an EEG to capture event-related desynchronization between the brain’s alpha and beta rhythms (111). Conceivably, information gathered from such experiments could be applied to Martel’s camera techniques in *La mujer* to study the “relational nature of cinematographic style and intersubjectivity” (116). One could also cognitively assess Vero’s experience. In line with Mark Solms’ adaptation of Karl Friston’s free energy principle, Vero’s trauma might plausibly be the product of disjunction between the homeostatic, relaxed state of free energy she experiences during her drive and that of the cortical-alarm-triggering crash (Solms, 2021, 195–196). It would also be possible to read the film through the lens of neurodivergence. In such a reading, Vero possesses an unidentified (or unmentioned) form of cognitive variation that neuro-normatively biased audiences fail to recognize and consequently misperceive as something “amiss” with her.

The confusion about what is going on only thickens when Vero attempts to obtain a concrete proof about what happened that day. Neither the hospital nor the local police have any record of a child’s death in a hit and run, although a young indigenous boy (one of the three in the opening) has died under disconcertingly unclear circumstances. When Vero confesses to her husband, he refuses to believe her. Returning with her to the crash site, he protests she only hit a dog before taking her car to be repaired. Her efforts to obtain her hospital admission and X-rays are likewise in vain: the hospital denies she was ever there. No X-rays can be found. The hotel lacks a record of her stay. By the end of the film, the only substantive change is to Vero’s hair color, which she re-dyes from blonde to brunette. Whether Vero has committed murder, who or what killed the indigenous boy, and if the opening scene was her deranged, hallucinatory projection or a real event, are left open questions. The film takes leave of them with the same indifferent shrug that Vero wanders away from the hospital intake paperwork.

This ambivalence encourages the spectator to regard the film’s central drama as a clash of objectivity and subjectivity. It appears to leave the spectator to hunt for clues as to what *really* happened or acquiesce to the idea that Vero’s perspective is sufficiently distorted as to make it impossible to know. Both her name and the film’s title reinforce this binary. In Spanish, Vero is related to “truth” (*la verdad*) and “sight” (*ver*, to see), making her character the embodiment of mechanical objectivity’s dictate that seeing is believing. “Sin cabeza” refers to the mental state of losing one’s bearings, sometimes due to love, but equally in instances of severe shock, as in the English language concept of “losing one’s head” (Martin, 2013, 145; Rodríguez, 2014, 98). Vero, the title seems to suggest, has gone out of her mind and been pursued by the camera into the twilight of her reason. It is the audience’s task to see the truth despite her unreliable narration.

At the same time, wariness about such willful guidance is apropos given Martel’s allusions to the Argentine Dirty War (1976-1983) throughout *La mujer* and her Salta Trilogy. These are the three films she created between 2001 and 2008 about the women of Salta. The *coup d’état* that overthrew the Perón regime in Argentina in 1976 brought to power a military dictatorship that, on the premise of purging political dissidents, relied on genocidal practices to systematically terrorize the population. Their actions made more than 30,000 people “disappear,” many of whose final fate and physical remains have yet to be recovered and may never be. The forcible disappearances were subsequently recognized by the Argentine judicial system as crimes against humanity. Known as *los desaparecidos* (the disappeared), they are marked as much by the absence that shrouds them as the complexity, if not impossibility, of mourning their loss.

The Salta Trilogy seldom refers to the Dirty War outright. Instead, it evokes the deceptive processes used by the dictatorship. The three films realize a feeling of heterotopic ghostliness and uncertainty rather than offering assurance that the era’s atrocities are well and truly past. Like its counterparts, *La mujer* cinematographically conjures the perceptual manipulations used by the dictatorship to identify the limitations of testimony. In place of a treatise on “what happened” according to those who “really saw it,” *La mujer* remains attentive to the peculiar mode of disavowal that sustained a 30,000-person disappearing act. As one recent monograph points out, *La mujer* is less concerned with objects and phenomena that can be immediately seen than with a White elite’s sanctioned blindness to the social, racial, and political ordering of society and unique forms of *not* seeing it are employed to ensure the continuity of preferable fictions (Gemünden, 2019). This sentiment is neatly summed up in Vero’s husband’s refrain: “*no pasó nada*” (nothing happened). In place of showing particular objects and events, then, Martel shows the array of socioeconomic, cultural, historical, and even scientific blockages that make them invisible. She shows strategies of ideological dis-apparition.

The film achieves this in two ways: by staging such blockages as physical barriers on screen and by framing shots in a highly unusual way. The barriers take the form of surfaces that interfere with the audience’s direct line of sight. They distort their object or yield only partial access to it by forcing the audience to peer through or around them. There are, for example, Vero’s lacquer black sunglasses, which make her face impossible to see until after the crash. There is her car windshield covered with a kaleidoscopic profusion of raindrops. The mirrors in her home. These surfaces help remind the viewers that the images they see are heavily mediated. They prompt the audience to recall that cinema is similarly contingent on making (in)visible and so posesses a structural resemblance to the *junta’s* actions in creating a simulacrum of reality. In place of an (impossible) declaration of censorship—impossible because it has also been censored—the onscreen barriers imply the existence of “evidence” that can only declare itself as a kind of erasure. They serve as formal cues that Vero is likely being systematically gaslit by a patriarchal regime complicit with, perhaps descendants of, the Dirty War’s military perpetrators.

Treating “sin cabeza” as a reference to a Vero’s putative loss of mental functioning (through trauma, neocolonial guilt, brain damage, or free energy disturbance) thus mistakes the socioeconomic and patriarchal construction of a normative femininity for Vero’s psychical interiority. Her interiority, however, is precisely what has been carefully whittled away. Even the fact that Vero embodies a normative fantasy cannot be stated outright: who would do the speaking? Unable to be expressed directly, her status as a depersonalized receptacle for gendered clichés is instead transferred to the film’s cinematography. Martel’s choice of camera placement and framing (her selection of what to show in a particular shot) indicates as much by repeatedly decapitating Vero through forcing her head off-screen. In the crash scene, for example Vero’s head vanishes from view at the moment of impact. Subsequent camera work similarly makes a point of dramatically cropping out her head. As she stands in the hospital’s X-ray room, the lowered arm of the X-ray machine blocks it from view. When she locks herself in the bathroom at home, the camera focuses on the doorframe and her partial reflection in the bathroom mirror, showing her only from the neck down. As with the reflective surfaces and Vero’s changing hair color (perhaps various shades of *verdad* on display?), the shots of Vero’s headless torso say almost nothing about what or whom is being represented. They say a great deal, however, about the forces conditioning her representation. The technique subtly conveys Vero’s status as an average signifier for a wishfully conceived woman. The cinematographic loss of the head expresses her deprivation of her individuality, a loss that “she” nevertheless cannot articulate precisely because of having been made a collection of platitudes.

The cinematography supports *La mujer’s* investment in a broader process of critical retraining. By exposing the constructed nature of evidence, Martel conjures the truth-bending practices of the *junta’s* cover-up and confronts her viewers with the need to find techniques for looking and thinking beyond canned narratives. Cinema, she argues in a 2012 interview, is better regarded as a tool to target unwittingly calcified perception and the onset of interpretive rigor mortis than it is a reflection of human perceptual activity.

I believe that the tools cinema has to communicate a perception of time and space allow us to question perception […] When perception is questioned, and in some ways cinema allows you to do it, the world reveals itself for brief moments. I think that’s what cinema is; more than revealing something about human beings, it reveals something about our perception. In that sense, it is very political, because if it helps us unlearn in a certain way of looking at things, then it might be possible to truly see certain things (Castanheira, 2012).

She explicates with reference to *La mujer:*

In *La mujer sin cabeza*, what interested me was to approach a mechanism that was superlatively used during the dictatorship that was essentially “We don’t realize what’s going on.” […] That “I was unaware of what was evident” mechanism was what I wanted to approach (Ibid).

In both statements Martel prompts her viewers to engage in acts of unlearning that distinguish between habitualized, mainstream perception and ways in which cinema trains the viewer to perceive (and so think) differently. Her fictitious film proves a better objective imaging device than both the X-ray machine it depicts and the disturbed first-person perception it appears to portray. In place of a portrait of psychological or cognitive distress, *La mujer* foregrounds new ways of looking at mental life by paying attention to strategies that make it visible.

The problem with neurocognitive readings of *La mujer* is thus not that they are implausible but that they are limited readings. By demanding that viewers engage exclusively on the level of appearance, interpretations stressing embodied simulation, Friston free energy, or neurodivergence awkwardly repeat the same erasure exemplified by the refrain “no pasó nada.” Nothing to see here. Paying lip service to empathy and intersubjectivity—whether *via* a biological basis for co-feeling or neurological difference—they mistake the capacity to perceive with the learned ability to interpret, invisibilizing the film’s cinematographic effort to disclose the production of evidence in the process.

This raises a number of difficult questions. Is the awareness of intersubjectivity gained through a cognitive reading comparable with that available by attending to the film’s meditation on a violent regime’s efforts to prevent its population from seeing too much and asking too many questions? How should one treat the fact that starting with the former largely blocks the latter? What role should history play in neurosciences? What to make of a focus on empathy that explains the pleasures of movie-going while perpetuating a tired narrative about female feeble-mindedness and the dispensability of indigenous bodies? How to understand interpretations that, by insisting “seeing is believing,” work in direct opposition to the film’s subtle efforts at indirect communication? Do neurocognitive analyses and humanities bids for Vero’s pathologization (trauma, amnesia, etc.) sustain the disappearance strategies the film seeks to divulge? While it is of course possible to explain Martel’s visual choices in *La mujer* in terms of what cognitive film theorists William Seeley and Noël Carroll call “variable framing” (a shift in camera position on emerging events in the movie world), doing so only reproduces the same problem the film seeks to subvert (Seeley and Carroll, 2014, p. 238). Seeley and Carroll maintain that viewers will always be *visually guided* to “critical story information.” Unfortunately, this position both conflates visual prominence with narrative significance and presupposes to know at the outset what the “critical information” is, thus short-circuiting the act of reading (240).

To return to Plato, the risk involves falling for a sophistic trap: mistaking illusion for reality in cases where illusion does not declare itself in bright capital letters. “Thus whoever says there exists a ψευδ ´ηςλóγ*o*ς is saying there is a letting be seen that conceals, or an opening up that occludes,” Heidegger notes in his lectures on the *Sophist* (Heidegger, 1997, p. 282). Failing to account for this problem is why well-meaning efforts to join critique (in the Kantian sense of *Kritik*) and neurosciences frequently come up short. *Neuroscience and Critique’s* (2016) admirable undertaking engages the Enlightenment tradition of *sapere aude* (dare to know!) without pausing to examine the self-produced limits within that very tradition.

## Discussion

### Project for a Humanist Psychology

One plausible move at this point would be to invoke Mark Solms’ neuropsychoanalysis as a means of integrating objectivity and subjectivity, and so linking the neurosciences and humanities. My proposal, however, is different from Solms’ ideas, as well as those of affective neuroscience, the study of how the brain generates emotions. I am suggesting the need for a new methodological approach, one engaging the neurosciences as part of the mimetic tradition that makes use of aesthetic objects as critical tools.

Solms’ work first took off in the late 1980s when he proposed an unexpected compatibility between Freud’s early neurological research and contemporary neuroscience on the basis of Freud’s “Project for a Scientific Psychology.” Solms suggested that psychoanalysis was a detour driven by Freud’s lack of access to modern neurobiological methods enabling the study of mental events (Solms and Saling, 1986). Had Freud possessed fMRI scans, discussions of the cortical fallacy might have emerged sooner, leaving little need for the descriptive vagaries of metapsychology. Motivated by studies showing a physiological basis for Freud’s work on dreams, as well as research demonstrating the neurobiological reality of affect (Antonio Damasio, Jaak Panksepp, and Oliver Sacks), Solms took his patients’ stories about their experiences seriously. He proceeded on grounds that such stories (traditionally excluded from neuroscientific data) shed light on real phenomena. “My emerging dream-research findings had convinced me that subjective reports had a vital role to play in neuropsychology,” he remarks (Solms, 2021, p. 30). He configured neuropsychoanalysis’ disciplinary bridge in terms of the neuroscientific viability of first-person narratives. “I have spent the last three decades […] trying to restore subjectivity to neuroscience,” he writes in his latest book (44).

This is an appealing link. It returns psychoanalysis and patient narratives to a place of prestige within positive knowledge and seems to offer a more humane, ethical, and listener-based clinical approach to suffering. Solms also repeatedly stresses the need to give Freud his due. He emphasizes his willingness to do so even in the face of warnings from colleagues about relating contemporary research with psychoanalytic pseudoscience. He makes a point of mapping psychoanalytic concepts onto testable reality, “translat[ing] such metapsychological insights into the language of anatomy and physiology” and correlating “Freud’s inferences about the functional mechanisms of subjectivity with their physiological equivalents” (Solms, 2021, pp. 35, 44). The research he conducted in partnership with British neuroscientist Karl Friston even led Solms to quantify the Freudian drive for first the time (177). In an article published in 2020, he resurrected Freud’s discarded “Project” and “completed it” by redefining Freud’s abbreviations (Q, Q ´η, φ, ψ, ω, W, V, and M) in light of contemporary neuroscientific knowledge (Solms, 2020).

Advocating for the neuroscientific validity of patient narratives is not the same as acknowledging a subjective dimension in the neurosciences, however. In fact, it is the opposite. Subjectivity in Solms’ model is valid only to the extent that it can be framed as not being subjective at all. In other words, patient stories become scientifically permissible by virtue of their amenability to empirical verification and/or connection with real physiological phenomena. Far from nuancing the objectivity-subjectivity binary, such work reaffirms it, insisting that subjective observations are viable only when they meet the standards of neuroscientific objectivity. Whence the priority given to quantifying the Freudian drive and “translating” psychoanalytic concepts into physiological equivalents. They would remain illegitimate otherwise. Presumably, in the absence of affective neuroscience’s demonstration that “emotions exist,” Solms’ patients’ accounts would also remain mere stories.

This leads affective neuroscience and the theories building on it to an exceptionally literalist hermeneutics. Because aesthetic objects are emotively inflected or because they contain thematic descriptions of psychological experience, such objects are thereby proof of neuroscientific claims. A few examples. In Jaak Panksepp’s breakthrough text *Affective Neuroscience* (Panksepp, 1998), an excerpt from Lev Tolstoy’s *The Kreutzer Sonata* (1890) introduces “Neural Control of Sexuality” because describing lust. A selection of poetry from Joan Walsh Anglund on love precedes “Love and the Social Bond.” In Damasio’s (2018) *The Strange Order of Things*, the poet Fernando Pessoa’s remark from *The Book of Disquiet* that “instruments grind and play away inside of me” prompts Damasio to offer Pessoa assistance in “identifying” the physiological correlates of this metaphorical orchestra. Pessoa’s stated inability to discern the “fiddlestrings and harps” sounding inside him (and “only [hear] the symphony”) apparently requires neuroscientific explication (101).

In the process, the matter of how representational mediation conditions the neurosciences gets lost. Tolstoy’s controversial portrait of female sexuality in *Kreutzer* (the woman in question is murdered by her jealous husband) vanishes without a trace. That Anglund is writing about love for a children’s book and that Pessoa’s excerpt is from the “factless autobiography” of fictitious alter-ego Bernardo Soares (not Pessoa as author) likewise disappear.^4^ With them, a host of related questions dissolves into the ether. Is Tolstoy’s presentation of a pathologically lustful femininity in *Kreuzer* naturalized by Panskepp’s epigraph and then inscribed within the “sociobiology of sexual attachment”? Does the shadow of this move return in the misplaced bid for gender equity on display in Solms’ fable of the “woman scientist” he calls “Eve Periaqueduct” in *The Hidden Spring*? Eve’s name and job as a structural engineer on a leaky dam in the story Solms misidentifies as a fable making up much of Chapter Eight, seamlessly reduce her to her reproductive biology (Solms, 2021).^5^ Does it matter that the Anglund poem offers a portrait of love sufficiently simplistic for a child audience? What does it mean to identify the physiological underpinnings of a fictional character’s metaphorical orchestra in any case?

The tendency toward literalism would be less problematic if it did not raise a tricky question about Freud’s “Project,” the text around which Solms’ justification for neuropsychoanalysis pivots. The issue involves knowing what Freud’s “Project” depicts. Solms repeatedly cites the opening lines of “Project,” in which Freud states he is seeking “to represent psychical processes as quantitatively determinate states of specifiable material particles.” Solms proposes that neuropsychoanalysis will make good on this abandoned but neuroscientifically promising endeavor (Solms, 2020, 2021). Freud’s references to “neurones” are the basis for Solms’ argument that Freud was a materialist after all, even if he subsequently went off track.

A great deal of ink has already been spilled by historians of science and philosophers on whether Solms’ position is historically compatible with Freud’s work and whether neuropsychoanalysis remains consistent with psychoanalysis (Bassiri, 2013; Guenther, 2015; Galgut, 2021). These works make a number of wonderful insights. They nevertheless neglect an interpretive analysis of “Project” and repeat Solms’ assumption that “neurones” mean physical cells. There is a case to be made, however, that Freud’s use of the term is metaphorical and that regarding it as such would benefit neuroscience.

The “Project” was actually a series of untitled, posthumously published letters to Wilhelm Fliess written between 1895 and 1896 and only designated “Project for a Scientific Psychology” by Freud’s English translator (Freud, 1987). In them, Freud’s bid to depict “psychical processes as quantitatively determinate states of specifiable material particles” is quickly undermined by his qualitative approach. There is a substantive disjunction between what Freud says he is doing and what takes place on the page. First, there are Freud’s symbols. The Greek letters and German abbreviations he uses seem mathematically rigorous but are quite ambiguous. Although Freud touts the need to work quantitatively, he does not make use of a single actual measurement in “Project.” “Φ, ψ, and ω” respectively designate systems of “permeable” (*durchlässige*) neurons, “impermeable” (*undurchlässige*) neurons, and neurons that when excited by perception give rise to “conscious sensations” (*bewusste Empfindungen*). Q is a quantity of magnitude in the external world, and the vaguer Q ´η is something like a quantity of psychical magnitude. To reiterate Freud never assigns concrete numerical values to Q or its variations; he simply states they have the quality of possessing quantity. Neither does he offer any means for measuring permeability. In fact, “Project” little resembles Gustav Fechner’s or Wilhelm Wundt’s psychophysical research, from which some of Freud’s terminology is nevertheless drawn. This makes it hard to know whether writing a “New Project for a Scientific Psychology” completes Freud’s text or entirely reimagines it (Solms, 2020).

Second, Freud never says that psychical processes are quantitatively determined states. He merely says that they can be represented as such. The German is *darstellen* (“to depict, to portray”). Freud’s goal is “to represent [*darzustellen*] psychical processes as quantitatively determinate states of specifiable material particles.” Solms ignores this qualifier, but it is an important, consistent feature of “Project.” Freud returns to *Darstellung* when discussing the possibility of representing memory and when specifying how to represent it. He writes about the idea of a *Darstellung* of the *Neurone* and entitles Part III, “Attempt to Represent [*darzustellen*] Normal ψ Processes” (Freud, 1966, p. 360). Taking “Project” at face value, however, Solms’ literalist reading focuses only on Freud’s seeming materialism. This misses “Project’s” similarities to Freud’s later, allegedly unscientific work. Such literalism is thus strangely insufficient, preferring a weak presentism to what Freud writes on the page.

Solms thereby neglects a reading of Freud arguably more pertinent to his endeavor because able to take account of subjectivity in neurosciences instead of objectivity by another name. If anything, “The Project for a Scientific Psychology,” inaugurates a humanist psychology. The letters mark the beginning of an evolving series of metaphors Freud will use across later publications to showcase different ways of approximating mental functioning. Writing just 3 years afterward, Freud comments in the *Interpretation of Dreams* (1899) that “we should picture [*vorstellen*] the instrument which carries out our mental functions as resembling [*wie etwa*, a bit like] a compound microscope or photographic apparatus, or something of the kind [*u.dgl*., or something similar]” (Freud, 1999, 541). As in “Project,” he calls on his readers to engage in an act of representation (the mental imaging of *Vorstellung* rather than the visual imaging of *Darstellung*), which is further extended by the requirement to consider this instrument as being “a bit like” (*wie etwa*) a compound microscope. He points out again that psychical locality cannot be shown directly. It is not the imaged object (the histological slide beneath the microscope, the numerical quantity) but only roughly analogous to the operations of the image-producing technology. This highlights the difficulty of picturing the mind. Is measurement, the microscope, photography, or something else “like” these technologies appropriate? In 1925, Freud proposed a further possibility: the mystic writing pad (*Wunderblock*). “A Note on the Mystic Writing Pad” suggests a popular children’s toy as an analogue. The toy, a kind of writing tablet, was made of a sheet of celluoid and one of wax paper positioned above a dark wax base. Children could easily make marks by applying a stylus to the sheets and just as easily erase the marks by lifting them. Freud argued the wax base resembled the unconscious insofar as it retained the impression of all previous marks, even those that had been erased. The top sheets were like consciousness insofar as they could be constantly make available for new impressions.^6^

These instances disclose how the selection of metaphor tacitly conditions the understanding of mental functioning. Freud’s representation of mental processes by way of the microscope, the camera, and the mystic writing pad anticipated the 1950s shift to the computer as the next iteration in the chain, followed by plasticity in the early 2000s. Of late, working models include the 4E approach (no less metaphorical for relying on abbreviations of four words beginning with the letter “e,” at least in English) and Friston free energy. This last comes in full circle by treating its own statistically determined picture of the mind-brain relationship as the basis for the future creation of an “artificially conscious mind,” which Solms speculates could be generated by “reverse-engineering the [natural] mind’s functional organization” (Solms, 2021, pp. 280, 282).

One could, by contrast, maintain that there is complementarity between psychoanalysis and the neurosciences simply by acknowledging the role of mimesis in Freud and Cajal’s research. In other words, one could recognize the play of *logos* and *pseudos* in both. Accepting that Cajal’s and Freud’s outwardly literal neurons are less mechanically objective than hitherto understood would not preclude neuropsychoanalysis, neuroimaging, statistical modeling efforts, or even a continental philosophy of the brain. It would merely introduce a perspective from which to consider their debt to representation. In addition, it would enable the neurosciences to engage aesthetic objects and psychoanalytic concepts as a productive site of tension. To those who doubt that working in this way is possible, I would point out that it has already been done. Art historian T. J. Clark’s remarkable reading of Freud’s “Project” through Paul Cézanne’s painting “The Large Bathers” (1898–1905) operates in just this manner (Clark, 1999).

Making the shift on a large scale, however, requires willingness to regard both aesthetic objects and psychoanalytic thought as valuable to the neurosciences in terms of their resistance, their *Widerstand*, to being subsumed beneath the neuroscientific umbrella. Because it necessitates the ability to read aesthetic objects and attend to language and rhetoric, however, the task cannot be left to intellectual historians or historians of science alone. This type of thinking requires skilled humanists with the courage to invest in the legitimacy of their own practices, even in the face of easier, more routinized epistemic justification for the humanities through the solaces of rational choice. Advocating for physics over metaphysics and psychology over metapsychology may make for clever prose when contrasting Freud’s psychoanalytic trajectory after “Project” with contemporary scientific research on the brain. At the same time, it naively imagines that the *pseudos* can be pruned away by lopping off the objectionable prefix “meta-.” Adopting a less binary approach to objectivity and subjectivity may seem (to some) to threaten a neuroscientific regression, a turn back into the darkness of the Platonic cave, something like consent to be duped. To paraphrase Rose and Abi-Rached (2013, p. 2), however, there is no reason for neurosciences to fear the humanities. Subjectivity has been a part of neuroscientific research from Cajal’s work onward.

## Data Availability Statement

The original contributions presented in this study are included in the article/supplementary material, further inquiries can be directed to the corresponding author.

## Author Contributions

The author confirms being the sole contributor of this work and has approved it for publication.

## Conflict of Interest

The author declares that the research was conducted in the absence of any commercial or financial relationships that could be construed as a potential conflict of interest. The reviewer DM declared a shared affiliation with the author to the handling editor at the time of review.

## Publisher’s Note

All claims expressed in this article are solely those of the authors and do not necessarily represent those of their affiliated organizations, or those of the publisher, the editors and the reviewers. Any product that may be evaluated in this article, or claim that may be made by its manufacturer, is not guaranteed or endorsed by the publisher.

## Footnotes

1 Freud, 1955, p. 536 [541]; Ramón y Cajal, 1996, p. 106 [139]; Münsterberg, 2002, p. 90. Brackets refer to original language pagination.

2 That the HBP lurched to a very public halt just 18 months after its heavily publicized kickoff, on the grounds it was wildly unrealistic, is as much a tribute to cognitive representation’s ongoing significance for the neurosciences as it is to its enduring capacity to provoke debate.

3 By his own admission, he was scientifically “devoted to the retina” (Ramón y Cajal, 1996, p. 576). Its similarity to photographic film was not lost on him. He remarks that the pigment rhodopsin in the retina’s rods “possesses the remarkable property of decomposing and turning pale under the action of light, like the silver bromide of a photographic plate” (Ramón y Cajal, 1899–1904, p. 591). “The Structure of the Optic Chiasm” (Ramón y Cajal, 1898) demonstrates his awareness that sight involved image distortion. In order to be reproduced as “mental projections,” external objects had to be disassembled, scrambled, and recreated fresh by stereoscopic vision. Cajal also used the photographic term “toning” (*viraje*) and the scientific term “staining” (*tincion*) interchangeably in scientific publications (Márquez, 2004, pp. 148–149). The swap makes sense given that Cajal relied on silver nitrate to develop both his photographs and his histological preparations. His book on color photography from 1912, *Fotografia de los colores*, was one of the first on the subject (Triarhou and del Cerro, 2008).

4 Soares, in any event, writes of his soul as a “hidden orchestra,” not one awaiting disclosure.

5 The chapter is supposed to illustrate predictive hierarchy in the nervous system. Eve, however, records “long-term leakage patterns” on the dam and is described as concluding her career in the local municipality with an inquiry about a “reproductive department” that could produce more dams.

6 On Freud’s metaphors, see Derrida (2001). I am unable to discuss psychic *écriture* here for reasons of length.
